# Supplementary material for: Are carbon emissions trading and green financial instruments synergistic? -Comprehensive quantitative research based on content analysis
Source: PLoS One. 2024 Mar 7;19(3):e0298601. doi: 10.1371/journal.pone.0298601 (PMC10919601; doi:10.1371/journal.pone.0298601)
Supplement: S1 Data — (ZIP) [file pone.0298601.s001.zip › Data/地级市Z_F工作报告.zip!/║╙─╧/╓▄┐┌╩╨/└┤╫╘╣1⁄2╓┌║┼í╛┬φ┐╦╩2╛▌═°í┐_2019.docx]

各位代表：

现在，我代表市人民政府，向大会报告工作，请予审议，并请各位政协委员和参加会议的同志们提出意见。

一、2018年工作回顾

过去的一年，面对严峻复杂的经济形势和艰巨繁重的发展任务，在市委的坚强领导下，全市上下深入学习贯彻习近平新时代中国特色社会主义思想和党的十九大精神，认真落实省委、省政府决策部署和省委书记王国生同志、省长陈润儿同志调研指导周口时的讲话精神，务实重干，砥砺奋进，经济社会发展保持了总体平稳、稳中有进的态势，实现了“三高三优”目标。全市生产总值增长8.2%，高于全省0.6个百分点；财政总收入增长21%，高于全省 9.1个百分点；居民人均可支配收入增长10.1%，高于全省1.2个百分点；二三产业占比83.3%，提升1.8个百分点；城镇化率提高1.6个百分点，增速居全省第1位；生态环境质量好于全省平均水平，完成了市四届人大三次会议确定的主要任务。

这一年，一些工作取得了突破性进展，主要表现在十个方面：

——城市建设有了新突破。中心城区谋划实施项目103个，总投资1315亿元，实现了大建设、大发展、大变化。全省第五次百城建设提质和文明城市创建工作推进会在我市召开。

——脱贫攻坚取得新进展。4.48万户、15.45万农村贫困人口脱贫，246个贫困村出列，沈丘县实现脱贫摘帽，6个拟退出的贫困县完成县级自评、市级初审、省级核查。

——临港经济开启新篇章。2018中国临港经济发展高峰论坛成功举办，周口港航集团组建运营，中心港6个港池主体工程即将完工，物流园区初见雏形，和润粮食深加工和物流仓储等一批产业项目落地建设，全市港口货物吞吐量达1300万吨，内河航运知名度不断提升。

——县域经济实现新跨越。7个县（市、区）生产总值超250亿元；5个县（市）一般公共预算收入超10亿元。2个产业集聚区晋升二星级，二星级产业集聚区达8个。

——返乡经济呈现新局面。新增返乡创业人员2.3万人、市场主体2.2万户，带动就业14.9万人。3家创业园区、8家返乡企业分别入选省农民工返乡创业示范园区和示范项目。郸城县被评为全国农村创业创新典型县。

——财税收入步入新阶段。一般公共预算收入完成129.3亿元，增长15.7 %；税收收入增长21.4%，占一般公共预算收入的70%，提高3.3个百分点；市本级一般公共预算收入29.4亿元，增长34.3%，增幅居全省第1位。同时，一般公共预算支出617.9亿元，增长20.4%，增幅居全省第1位。

——招商引资迈出新步伐。新签约亿元以上项目289个，总投资2455.7亿元。恒大集团将投资建设周口中央商务区、周口职业技术学院合作办学专升本等项目。

——重大赛事展现新形象。成功承办省第十三届运动会和省第七届残运会，并取得了省十三届运动会金牌总数全省第二的好成绩。

——教育工作再创新佳绩。项城、沈丘通过义务教育发展基本均衡县国家评估认定。高考本科上线人数3.8万人，上线率50 %，居全省前列，全省理科第1名花落周口。周口科技职业学院成功升本，我市新增1所本科院校。

——饮水安全得到新改善。中心城区实现南水北调供水全覆盖，50万居民喝上丹江水。解决了淮阳、郸城、商水、西华85万农村居民的饮水问题，在全省率先实现村村通自来水。

一年来，重点做了以下工作：

（一）抓结构调整促转型。三次产业结构调整为16.7‥45.1‥38.2。粮食总产达180.38亿斤，再创历史新高。优质小麦、优质大豆、优质花生种植面积分别增加42万亩、15万亩、41万亩，玉米调减15万亩。新增新型农业经营主体4170家。实施技术改造项目308个，启动智能化改造项目28个。规模以上工业增加值增长8.2 %，高于全省1个百分点；高技术产业、高成长性制造业增速分别快于全市工业1个百分点、2.8个百分点。社会消费品零售总额增长10.7%，高于全省0.4个百分点。电子商务交易额354亿元，旅游综合收入171.7亿元，商品房成交额294亿元，分别增长17 %、12.9 %、37.2 %。服务业增加值增长10.8 %，对经济增长的贡献率达44.6%。

（二）抓城市建设提品质。城市规划体系进一步完善，“两违”治理逐步实现常态化。棚户区改造步伐加快，中心城区完成征迁3.8万户、1300多万平方米，开工建设安置房6.3万套。水系综合治理、高铁站等一批重大项目顺利推进。森林公园、铁路主题公园、植物园改造提升3个项目的一期工程，均实现当年建设当年开园。引黄调蓄工程3600亩湖面、建业6000亩绿色基地初具规模。40个绿地游园建成开放。文昌大道等40条主次干道和4个高速公路出入市口完成升级改造。实施亮化、净化、美化工程，“五城联创”深入推进，城市环境持续优化。万科、上海世茂等一批项目纷纷进驻，投资100亿元的2个万达商业综合体项目开工建设，红星美凯龙等商贸综合体建成投用。项城、淮阳被评为创建全国文明城市提名城市，鹿邑成功创建国家级园林县城，太康、西华成功创建省级园林县城。

（三）抓三大攻坚补短板。在防范风险上，金融运行平稳，政府债务总体可控，一批问题企业、问题楼盘、问题工程得到妥善处置，“温比亚”台风等自然灾害得到有效防控，安全生产和食品药品安全形势平稳向好，信访总量持续下降，社会大局保持和谐稳定。在精准脱贫上，坚持智志双扶“双轮驱动”，实施项目2128个，其中产业扶贫项目1468个，辐射带动贫困人口23.9万人，探索出“两保两带”模式和特困群体“五养”模式，郸城、太康在全国会议上介绍典型经验，商水“巧媳妇工程”被中央级主流媒体报道。在污染防治上，狠抓“六控”措施落实，PM2.5、PM10平均浓度分别为58微克/立方米、103微克/立方米，优良天数220天；落实“河长制”，11个地表水考核断面水质平均达标率 93.6%，城市集中式饮用水水源地水质达标率100%。完成造林14.3万亩，创历史新高。

（四）抓动能转换增活力。国企改革扎实推进，处置“僵尸企业”16家。“放管服”改革力度加大，取消行政审批事项9项、证明事项281项，“三级十同”事项“网上可办”“一网通办”实现率均达100%，“互联网+政务服务”建设走在全省前列。财税、金融、土地等领域改革稳步推进。出台推动工业高质量发展12条意见和扶持非公有制经济健康发展等一揽子政策措施，2300户个体工商户转为小微企业，100家小微企业升级为规上企业。乐普药业入选省百名非公经济企业。创建国家级企业技术中心4家、国家地方联合工程实验室3家，新建省级工程技术研究中心7家，新增高新技术企业24家、省长质量奖企业1家。5项成果荣获省科技进步奖，专利申请量增长28.1%。规模以上工业企业利润总额增长35.1%，高于全省10.6个百分点；工业增值税增长52.3%，增速居全省第1位。外贸进出口增长21.6%，高于全省16.3个百分点。固定资产投资增长9.7%，高于全省1.6个百分点。57个省重点项目完成投资385.4亿元，占年度投资计划的106%；313个市重点项目完成投资1237.5亿元，占年度投资计划的107.8%。

（五）抓民生改善添福祉。财政民生支出496.3亿元，占一般公共预算支出的80.3%。市重点民生实事和中心城区十件民生实事基本完成。城镇就业新增8.95万人，农村劳动力转移就业新增5.96万人。新建改建幼儿园100所，新增城镇义务教育学位2万多个。免费为城乡居民提供13类48项基本公共卫生服务。新建标准化卫生室97个，新增三级医院1家。新建村级综合文化中心3779个。市博物馆晋级为国家二级博物馆，关帝庙景区创建为国家4A级旅游景区。新建改建农村公路2291公里，拆除韭园、大李庄2个普通公路收费站。城乡居民基础养老金年人均增资216元，低保、优抚、残疾人“两项补贴”等政策全面落实。改造农村电网4612公里，改造农村危房1.8万户，改造农村厕所19.5万座。“六村共建”得到加强，村容村貌有效改观。“扫黑除恶”等专项斗争深入推进，现行命案侦破率100%，刑事案件明显下降，人民群众获得感、幸福感、安全感持续增强。

国防教育深入开展，国防后备力量得到加强，民兵调整改革任务完成较好，驻周解放军、武警部队、预备役部队、民兵为周口发展作出新贡献，双拥工作持续优化。民族宗教工作创新推进。妇女儿童、残疾人、红十字、慈善等事业健康发展。统计、海关、公积金、史志、档案、气象、地震、人民防空、外事侨务、社科研究、应急救援、援疆等各项工作均取得新成绩。

一年来，我们全面执行市人大及其常委会的决议和决定，自觉接受人大工作监督、法律监督和政协民主监督，认真办理人大代表议案、建议和政协提案，支持民主党派、工商联、无党派人士参政议政。切实加强政府自身建设，推进依法行政，驰而不息纠正“四风”，党风廉政建设深入推进，政府的治理能力和水平正在提升。

各位代表！

过去的一年，是全市上下担当作为的一年，也是以党的建设高质量推动经济发展高质量取得初步成效的一年。成绩来之不易。这是习近平新时代中国特色社会主义思想科学指引的结果，是省委、省政府和市委正确领导的结果，是人大工作监督、法律监督和政协民主监督的结果，是全市广大干部群众团结拼搏的结果。在此，我代表市人民政府，向奋战在全市各行各业的广大劳动者，向各级人大代表、政协委员，向各民主党派、工商联、无党派人士、各人民团体和离退休老同志，向中央、河南省驻周单位的同志们和驻周部队指战员、武警官兵、消防救援人员，向所有关心、支持、参与周口建设和发展的朋友们，表示衷心的感谢和崇高的敬意！

在看到成绩的同时，我们也清醒认识到，经济社会发展中还面临不少困难和问题。一是产业结构矛盾突出。农业比重大，优质农产品供给不足，现代服务业和高新技术产业占比偏低。二是新动能培育不足。新技术、新产业、新业态、新模式培育不够，创新型企业不多、体量较小。尤其是，研究与试验发展经费投入强度占规模以上工业增加值比重明显偏低。三是企业经营困难较多。融资渠道收窄，能源原材料、人工成本较高，环保、土地等制约因素增多，部分企业经营困难加重。四是民生领域存在短板。还有10.33万贫困人口尚未脱贫，环境治理压力较大，公共基础设施欠账较多，大班额、就医难等问题还比较突出，城镇化率分别低于全国、全省16.76个、8.89个百分点。同时，受土地、资金等因素制约，个别项目论证不充分，周（口）太（康）快速通道、人行过街天桥等项目没有启动，还有21个省市项目没有完成时序进度。政府个别工作人员存在思想解放不够、工作标准不高、落实效果不好等问题。为此，我代表市人民政府向全市人民表示深深的歉意！下一步，我们将高度重视、科学论证、精准施策、切实解决，努力把各项工作做得更好，决不辜负人民期待！

二、2019年工作安排

今年是新中国成立70周年，是全面建成小康社会的关键之年。做好政府工作，要以习近平新时代中国特色社会主义思想为指导，全面贯彻党的十九大和中央经济工作会议、省委十届八次全会、市委四届八次全会精神，统筹推进“五位一体”总体布局，协调推进“四个全面”战略布局，坚持稳中求进工作总基调，坚持新发展理念，坚持推动高质量发展，坚持深化市场化改革、扩大高水平开放，坚持以供给侧结构性改革为主线，贯彻巩固、增强、提升、畅通的方针，围绕“坚决打好打赢三大攻坚战、如期全面建成小康社会”的总体目标和“三高三优”工作目标，统筹推进稳增长、促改革、调结构、惠民生、防风险、保稳定工作，增强人民群众获得感、幸福感、安全感，保持经济持续健康发展和社会大局稳定，务实重干、敢于担当、加压奋进、跨越发展，为全面建成小康社会收官打下决定性基础，在中原更加出彩中绘就周口特色添彩画卷，以优异成绩庆祝中华人民共和国成立70周年。

今年经济社会发展主要预期目标是：继续保持“三高三优”，生产总值增长7.5%以上，全员劳动生产率增长7%，一般公共预算收入增长8%，税收占一般公共预算收入的比重达到70%以上，固定资产投资增长9%以上，社会消费品零售总额增长10%以上，居民消费价格涨幅3%左右，经济结构进一步优化，质量效益明显提升。创新发展要取得新成果，研究与试验发展经费投入强度达到1.5%左右，高技术产业、战略性新兴产业增加值占规模以上工业增加值的比重分别达到13%以上和20%左右。协调发展要取得新成效，常住人口城镇化率提高1.6个百分点以上，九年义务教育巩固率达到95%，医疗卫生、文化、体育等社会事业全面进步。绿色发展要取得新提升，单位生产总值能耗下降3%以上，主要污染物排放量完成省定目标。开放发展要取得新突破，进出口保持平稳增长，营商环境在全省评价排序中保持中上游。共享发展要取得新进步，居民人均可支配收入增长8%以上，5.3万农村贫困人口脱贫，城镇新增就业6万人以上，城镇调查失业率和城镇登记失业率分别控制在5.5%、4%以内。以上目标，更加凸显了新发展理念和高质量发展要求，与往年相比，增加和单列了创新、协调、绿色、开放、共享五个方面的内容，有利于引导各级各部门把工作重点放在推进高质量发展上来。

做好今年政府工作，完成上述目标任务，不仅需要我们把高质量发展指挥棒发挥好，推动新发展理念落地生根、开花结果，而且需要我们认清形势、把握机遇，凝聚共识、发挥优势。当前，不确定性因素增多，经济下行压力加大。但挑战总是与机遇并存，我们更要善于观大局、谋大事，积极应对、化危为机。从政策机遇看，近期，中央为应对国际形势，妥善处理内外需走弱、实体经济困难和金融风险，进一步加大改革开放力度，实施积极的财政政策和稳健的货币政策，相继推出了基础设施领域补短板、支持非公有制经济发展等一系列利好政策。淮河生态经济带上升为国家战略，省委、省政府全面铺开百城提质，联动实施引江济淮和赵口灌区二期等工程。这些都给我们带来了更多更大的发展机遇。从现实基础看，近年来我们实施了一大批项目，随着项目的投用，拉动效益凸显。去年以来，我们开展了大规模征迁，腾退近万亩土地，随着淮阳撤县设区的推进，城市空间拉大。今年郑合高铁开通，我们将迈入高铁时代，开放的步伐加快。目前，我们发展多式联运，打造豫货出海口，临港经济正成为周口发展的新引擎。伴随着大批返乡人士回归创业，返乡经济正成为新的增长极。我市有1000多万人口的市场消费潜力、较大的城镇化提升空间，必将释放出巨大消费和投资需求。同时，我们还应看到，当前全市上下人心思干、干事创业的氛围浓厚，加之恒大集团投身于周口建设，必将推动周口加速发展。综合研判，我们仍处于大有可为的重要战略机遇期，只要提振信心、保持定力、苦干实干，就一定能够实现新突破、开创新局面、再创新辉煌！

新形势新目标新使命，新征程新担当新作为。按照市委四届八次全会部署，今年政府工作总体上仍然是，持续推进“四大攻坚”，奋力实现“四大突破”，并根据现阶段的特点，着力做好以下工作：

（一）突出一个重点：项目建设。坚持把项目建设作为经济发展的重中之重，贯穿工作始终，千方百计扩投资、促增长。

抓进度。紧盯53个省重点项目和319个市重点项目，突出120个中心城区项目，着力抓开工、抓形象、抓投产，努力形成开工项目铺天盖地、在建项目热火朝天、竣工项目接二连三的生动局面。开工建设天虹纺织科技园等产业项目。加快建设万达等城市综合体项目。加快推进周口民用支线机场项目前期工作和西华、鹿邑通用机场，以及郑合高铁、三洋铁路、阳新高速周口段等基础设施项目。改造升级一批干线公路，创建一批“四好农村路”。加快推进西华、太康、郸城、沈丘等生活垃圾焚烧发电等能源项目。抓招商。落实国家、省出台的利用外资政策，鼓励支持各县（市、区）制定招商引资优惠政策，务求招商引资取得新突破。围绕主导产业，依托中央商务区、物流产业园等园区及产业集聚区、服务业“两区”等招商平台，突出补链延链强链等产业链招商，谋划确定200个重点招商项目，针对性地开展上门招商、精准招商，引进一批国内外知名龙头型企业落地，提高项目履约开工率。抓对接。加大对中央和省预算内投资项目、专项建设基金项目及专项债券的争取力度，引导金融机构加大对重点产业、重点项目支持力度。规划建设教育园区、文化创意产业园、健康养老产业园及静脉产业园等园区。抓服务。建立重大项目服务领导小组，严格落实分包责任制，推行从规划选址、手续办理到投产达效等全过程跟踪服务，统筹解决项目建设中遇到的审批和土地、资金等方面问题，确保项目早落地、早建设、早见效。

（二）实施“两项工程”：百城建设提质工程和乡村振兴工程。坚持突出重点，注重统筹，加快推动城乡大发展、大融合。

全面开展百城建设提质。坚持以水润城、以绿荫城、以文化城、以业兴城，全面铺开百城提质工程，深入推进“五城联创”，加速“中原港城”建设，推动城市高质量发展。加快完善规划。新纳入百城提质的县抓紧启动市政基础设施和公共服务设施、生态修复等专项规划的编制。中心城区、项城、扶沟上半年完成城市总体设计及城市中心区、滨水区域等重点区域的规划设计。推进国土空间规划系统建设，完成市、县国土空间规划编制工作。统筹中心城区和淮阳、商水、项城、西华规划融合。加快设施建设。启动实施高铁站至淮阳城市道路、中州大道北延至西华等项目。加快实施新北环、开元大道东连西延，尽快形成中心城区大环城交通格局。加快推进汉阳路升级改造、中原路跨沙颍河大桥等项目，实施富民路等断头路打通工程，八一路南通商水、文昌大道东延建成通车。推动中心城区7条水系治理工程和河西、东区、沙南3个污水处理厂项目建设。加快沙颍河景观二期、森林公园二期、铁路主题公园二期、植物园改造提升二期、建业绿色基地、沙河湾湿地公园等项目建设，60座绿地游园建成开放。完善高铁片区站台、广场、公交场站和路网等配套设施，确保按时满足通车要求。抓好老旧小区和背街小巷改造，建设一批停车场、公厕、垃圾中转站、公交充电场站等公共服务设施。加快开发经营。加快推进引黄调蓄工程等项目建设。积极与恒大集团对接，争取中央商务区等项目早落地、早开工。招引品牌企业入驻高铁片区，建设商业综合体。加大土地收储、出让力度，适度放开城市公益性、经营性设施，激发市场活力。持续实施棚户区改造三年计划，中心城区年内完成1.1万户、325.8万平方米征迁任务，启动实施8个安置区、1.4万套安置房建设任务。加快推进贾鲁嘉苑、建设嘉苑等已开工安置小区项目建设，让征迁群众尽早住上房、住好房。积极谋划新开工项目，确保8月底前全市33个项目、6.5万套安置房全部开工。统筹推进安置区内学校、卫生服务机构、便民服务中心等配套设施建设。提升管理水平。全面抓好城市环境治污、交通秩序治堵、市容环境治脏、公共服务治差“四治”工作，推行环卫保洁作业服务外包模式，逐步实现小区管理物业化、街道管理网格化、市场管理规范化、路面清扫机械化，力争国家卫生城市创建成功。继续保持对“两违”的高压态势，对存量“两违”分期分批拆除，对新增“两违”发现一起、拆除一起。同时，支持各县（市）加快“城市双修”，完善基础设施和公共服务体系。加强规划指导和政策扶持，着手打造一批历史文化名镇、旅游名镇和特色小镇。提升乡镇政府驻地建设、管理水平，着力改善市容市貌。加快区划调整步伐，认真落实户籍改革政策，促进房地产市场平稳健康发展，推动农业转移人口市民化，提升新型城镇化水平。

大力实施乡村振兴战略。认真贯彻落实习近平总书记在参加十三届全国人大二次会议河南代表团审议时的重要讲话精神，坚持农业农村优先发展，强力推动农业强、农村美、农民富。科学编制规划。坚持统筹规划，有效结合产业转型、人口分布、公共服务等因素，精准研判港城融合、城镇化发展等新趋势，科学编制市、县、乡三级乡村振兴战略规划，让乡村发展有规可依、有章可循。稳定粮食产能。扛牢保障粮食安全责任，加快建设粮食生产功能区和重要农产品生产保护区，推动藏粮于地、藏粮于技，确保粮食播种面积稳定在1000万亩左右、总产量稳定在160亿斤以上。加快高标准农田建设，每县打造一个10万亩的现代农业示范区。强化产业支撑。坚持质量兴农、绿色兴农、品牌兴农，大力发展生态农业、精深加工业和绿色食品业，延伸产业链、提升价值链、打造供应链，不断壮大金丹、普爱、雪荣、益海、牧原、天豫等产业化龙头企业，培育建设一批现代农业产业化集群。积极推进以郸城为核心区的国家农业高新技术产业示范区创建工作，打造周口农业品牌。推动农科院科研基地外迁，提升研发水平和带动能力。抓好返乡经济。返乡经济是周口加快发展的发力点。发挥我市在外务工人员多、成功人士多的优势，全面落实扶持政策，积极搭建融资、孵化、项目等平台，大力实施“回归”工程，吸引更多在外的人才、资金、项目返乡。鼓励返乡人员把先进的市场意识、科学技术和经营理念植入农村，培育新型农民，发展现代农业，让农产品种得好卖得好，推动农业提质增效。支持返乡人员兴办企业，培育一批新型企业、家庭农场等市场主体，让更多农民在家门口就业增收。深化农村改革。加快农村集体产权制度改革，完成农村集体资产清产核资，发展新型集体经济。深化农村金融改革，加快发展普惠金融、科技金融、绿色金融，强化农业保险保障作用。探索盘活农村闲置宅基地和农房，增加农民财产性收入。建设美丽乡村。实施“千村示范、万村整治”工程，深入开展“六村共建”，加快推进“厕所革命”，抓好垃圾污水治理、废弃物资源化利用、村容村貌提升，努力打造“四美乡村”和“五美庭院”。开展“万村通客车”提升工程，实施“农村公路通村入组”工程，加快乡村物流、宽带网络等基础设施建设。扎实开展全域国土综合整治，深入推进“大棚房”与农地非农化问题治理，严守耕地保护红线。深入开展农村精神文明创建活动，培育文明乡风、良好家风、淳朴民风。保护农耕文化、文物古迹、传统村落、传统建筑，留住乡愁。

（三）打好三大攻坚战：防范化解重大风险攻坚战、精准脱贫攻坚战和污染防治攻坚战。坚持目标导向和问题导向，抓重点、补短板、强弱项，坚决落实好各项措施，务求取得实效。

防范化解风险。坚持底线思维，着力防范各领域重大风险。政府债务方面，全面规范地方政府举债行为，坚决遏制增量，稳妥化解存量。做好土地储备债、棚改债等专项债的额度争取、统筹使用。鼓励通过市场化运作、区域土地平衡、项目融资与收益自求平衡等方式，提高资金使用效益，破解投融资难题。金融方面，严厉打击非法集资，加大陈案攻坚力度，遏制新发案件；探索开展民营企业债转股，抓好企业间担保链风险防范；高度关注金融机构不良贷款，严厉打击恶意逃废债等行为，坚决守住不发生区域性、系统性金融风险的底线。其他方面，压实安全生产责任，全面推行双重预防体系建设，健全应急管理体系，强化源头管控治理，坚决防范遏制重特大事故发生；增强防洪抗灾能力，防范自然灾害风险；做好突发公共事件预防处置，防范社会风险。

推进精准脱贫。当前，脱贫攻坚工作到了啃“硬骨头”的关键时期，抓住关键人、抓牢关键事、抓好关键点，确保6个贫困县如期摘帽。在问题整改上，对照中央和省市巡视巡察、督查暗访、核查调研中发现的问题，逐一对照过滤，确保即知即改、立行立改。在精准帮扶上，坚持因村因户因人施策，注重激发内生动力，采取以工代赈、生产奖补、劳务补助、开发公益性岗位等方式，充分调动贫困群众的积极性和参与度。突出产业扶贫，抓好标准化“扶贫车间”、产业扶贫基地等产业项目建设，带动更多贫困户就业增收；对市级产业扶贫基地开展“回头看”活动，确保贫困户年内覆盖项目2个以上，户均增收3000元以上。打好金融扶贫、产业扶贫保险保障“组合拳”，进一步落实行业扶贫政策，实现贫困人口产业帮扶和到户增收措施全覆盖。在贫困退出上，严格贫困退出标准和程序，做到脱真贫、真脱贫，确保脱贫成果经得起检验。对摘帽贫困县、贫困村和脱贫人口，在攻坚期内相关扶贫政策保持稳定，做到摘帽不摘责任、摘帽不摘政策、摘帽不摘帮扶、摘帽不摘监管。

抓好污染防治。深入实施经济结构提质、生态功能提升、国土绿化提速、环境治理提效“四大行动”，着力打造天蓝、水净、地绿的美丽周口。坚决打赢蓝天保卫战。狠抓能源消费结构、区域产业结构、交通运输结构等调整优化，持续抓好“六控”，坚决治理“散乱污”企业，建立覆盖所有固定污染源的企业排放许可制度。严格工地、道路扬尘管控，提高城市清洁标准，积极做好秸秆禁烧和重污染天气应对等工作，让人们享受更多蓝天白云。全面打好碧水保卫战。持续推进城市黑臭水体治理，年内中心城区基本完成消除黑臭水体整治工作，项城、扶沟基本消除黑臭水体现象，其余县城基本完成黑臭水体截污纳管、排污口整治等任务。加强重点流域水质和饮用水水源地保护，确保11个地表水考核断面达到省定目标值，城市集中式饮用水源地水质达标率98%以上。全面落实“河长制”，实施“四水共治”，加快贯通水系畅通水网，注重雨水、洪水、废水的资源化利用，启动区域生态治理和景观提升工程，努力实现河畅、水清、岸绿、景美。扎实推进净土保卫战。加强农业面源污染防治和固体废物处理，做好化肥农药减量增效等工作，推进受污染耕地安全利用和治理修复。加快推进森林周口建设，创建国家森林城市，完成造林25万亩，确保全国绿化模范城市创建成功。

（四）做好“四篇文章”：临港经济文章、产业转型文章、改革开放文章和社会民生文章。这是高质量发展的重要动力源和最终落脚点，必须聚焦聚力，抓住关键，以点带面，整体推进。

发展临港经济。临港经济是周口转型发展的突破点。树牢“临河就是临海、腹地也是前沿”的理念，以创建国家级多式联运枢纽试点城市为抓手，大力发展临港经济，带动更多产业、吸引更多要素集聚。修编规划。围绕淮河生态经济带建设，进一步完善临港产业发展规划、多式联运发展总体规划，对全市域、全流域的港口统一规划，实施错位发展；对沙颍河进行规划设计，将其打造成黄金水道、产业大道和生态廊道，形成周口特色发展黄金轴线。建好设施。加快建设大广高速港区互通立交项目，做好临港铁路专用线规划设计和项目前期工作。加快推进沙颍河航道升级改造、引江济淮工程周口段建设，中心港余下的6个港池35个千吨级泊位开港运营，着力打造公铁水多式联运示范工程。启动实施（B型）保税物流中心项目，完善提升口岸功能。高标准建设集装箱、钢材等专用码头以及锚地、堆场、岸电设施，提高配套设施的现代化水平。组装项目。加快推进豫东LNG应急储备中心、粮食物流园等项目建设。开工建设装配式建筑、创客空间及企业孵化器和沈丘港口物流园、商水临港产业园等项目。精心办好2019临港经济高质量发展论坛，力争引进一批临港产业项目。培育业态。重点发展集装箱物流、大件物流、冷链物流，进一步深化与郑州航空港、中欧班列、海河联运港际合作联盟对接合作，吸引更多大宗货物运量向周口集聚。积极培育一批建筑装配、电子信息、新能源等新兴产业。

推动产业转型。着眼高端化、智能化、绿色化、融合化发展目标，紧盯装备制造、现代物流等11个重点产业，统筹新兴产业培育和传统产业改造，加快转型升级步伐。壮大工业实力。工业经济是周口高质量发展的支撑点。加快产业集群培育，持续打造食品加工、纺织服装、生物医药3个千亿级产业集群，装备制造、电子信息、新型建材3个500亿级产业集群。着力推进六大主导产业转型升级，支持一批支撑带动能力强、关联度高的项目和企业做优做强。实施300个技术改造项目和20个智能化改造项目，创建2家智能工厂、4个智能车间、1个省级绿色工厂。加快培育智能装备制造、生物医药等战略性新兴产业，推动瑞特电气、乐普药业等企业快成长、上规模。引导企业增加研发投入，实施创新龙头企业提升引领、高新技术企业倍增、“科技小巨人”企业培育工程，新认定1个省级技术创新示范企业、2个省级工业公共研发设计中心。开展质量提升和对标达标专项行动，弘扬劳模精神和工匠精神，支持企业增品种、提品质、创品牌，打造“百年老店”。各县（市、区）明确产业主攻方向，强力推进“一县一主业”集群发展计划，优化资源配置，推动错位发展。聚焦产业集聚区二次创业，完善基础设施，提升园区功能品质，打造产业聚集、企业聚集、人才聚集的产业生态圈。支持金丹乳酸等企业上市、邦杰食品等企业新三板挂牌。支持建成区内企业退城入园。持续抓好产销、银企、用工、产学研“四项对接”，针对性解决企业发展中遇到的具体困难和问题。引导提升企业素质，促进二次创业和转型发展。提升服务业水平。加快传统服务业升级，改造提升老旧市场，规范建设农贸市场和小吃市场，引进便民店进社区，促进商贸业繁荣。大力发展现代服务业，加快培育家政、教育、育幼、养老等生活性服务业，提升产品质量，改善消费环境，让老百姓放心消费；引进一批工业设计、信息服务、检验检测等生产性服务业项目，拓展工业产业链和价值链。积极发展文化旅游业，在确保文物安全的基础上，保护开发好具有历史价值的老街区和历史古迹，让古老的文物活起来；依托沙颍河、淮阳龙湖、森林公园等资源，开发水上旅游和生态旅游。支持民间博物馆、收藏馆、艺术馆、非遗传承所等机构的培育建设，全面提升产业项目的建设标准和文化内涵。鼓励网购，发展健康护理、体育健身、绿色消费等新型消费业态。积极引进和培育龙头电商，全面推进电子商务进农村、进社区、进企业。发展数字经济，加快推进与浪潮集团合作，启动建设周口大数据产业园。调优农业经济。实施高效种养业转型升级行动，合理调整粮经饲结构，推进农业由增产导向转向提质导向，着力打造160万亩优质专用小麦、200万亩优质大豆、180万亩优质花生、200万亩优质果蔬、10万亩优质草畜生产基地；持续创建省级畜产品质量安全市，大力推进畜禽养殖标准化建设，加快畜牧业提质增效。推动融合升级。加快互联网在一二三产业等领域应用，鼓励引导有条件的企业向智能化、数字化方向发展，推动产业链再造和价值链提升。利用“制造+服务”这个转型的引领点，通过提供个性化定制、全方位售后等服务增值活动，增加产品附加值，提升企业盈利空间。

深化改革开放。加大力度，加快步伐，充分释放发展动能。坚定不移推进改革。全力打好国企改革“扫荡战”，在加快“僵尸企业”处置后续工作的同时，持续深化企业产权结构、组织结构、治理结构改革，加强和完善国有资产监管，积极稳妥推进混合所有制改革，做强做优做大国有资本。持续深化“放管服”改革，注重压环节、减时限、提效率，着力实现线上“一网通办”、线下“只进一扇门”、现场办理“最多跑一次”，有效解决企业和群众反映强烈的办事难、办事慢、办事繁问题。全面落实减税降费政策，深化增值税改革，将制造业等行业现行16%的税率降至13%，将交通运输业、建筑业等行业现行10%的税率降至9%，采取对生产、生活性服务业增加税收抵扣等配套措施，让所有行业税负只减不增；在城镇职工基本养老保险征收体制改革过程中，不增加小微企业实际缴费负担，不得自行对历史欠费进行集中清缴。打好优化发展环境“组合拳”，启动营商环境评价，积极开展营商环境核心指标对标行动、营商环境重点领域提升行动；加快诚信体系建设，坚决纠正一些地方和部门“新官不理旧账”的违约做法。落实好金融服务实体经济的相关政策措施，用好信贷、债券、股权“三支箭”，督促金融机构加大对民营企业、中小微企业的支持力度。扎实推进政府系统机构改革，统筹推进财政、供销社、农场、林权、教育、医疗、环保、城市综合执法等领域改革。全面提升开放水平。抓住高铁开通带来的机遇，主动靠拢、充分接受郑州的辐射和转移，对接“陆上丝绸之路”；利用沙颍河通江达海内河航运优势，打通周口和长三角等沿海地区的要素通道，对接“海上丝绸之路”；开工建设跨境电商创新创业孵化园等项目，加强与阿里巴巴等第三方大型跨境电商平台的对接，扩大“网上丝绸之路”的辐射面，努力让周口走在全省开放的前沿。加强对20家重点企业和重点进出口商品的监控和跟踪服务，保持进出口持续稳定增长。

改善社会民生。坚持以人民为中心的发展思想，挤出更多财力保障基本民生，扎实办好省市重点民生实事和中心城区十件民生实事，让人民群众有更多的获得感、幸福感、安全感。促进就业创业。落实就业创业扶持政策，突出做好高校毕业生、农村转移劳动力、城镇困难人员、退役军人等群体就业工作，鼓励支持外出务工人员返乡创业。

加强社会保障。将全民参保登记工作与扩大社保覆盖面有机结合起来，引导各类人员应保尽保。继续提高城乡居民基础养老金最低标准，继续调增企业和机关事业单位退休人员基本养老金。提高基本医保、大病保险、困难群众大病补充医疗保险的保障水平，落实80岁以上老人高龄津贴、城乡低保、特困供养、孤儿养育、残疾人“两项补贴”等政策。全面完成“四类重点对象”农村危房改造。发展社会事业。规划建设一批学校、医院，满足群众就近上学、看病。持续实施学前教育三年行动计划，加大普惠性幼儿园建设力度。抓紧实施农村寄宿制标准化学校建设、城镇学校消除大班额和教育信息化三大工程，力争太康、淮阳、西华、鹿邑和川汇区通过义务教育发展基本均衡县国家评估认定。继续实施中心城区教育振兴工程，持续打造高中教育品牌。大力发展现代职业教育，扎实开展“职业教育提升年”活动，提高职业教育服务经济发展的支撑能力。积极推进周口职业技术学院与恒大合作办学专升本项目，支持升本后的周口科技职业学院加快发展，支持周口高级技工学校晋升技师学院、联营职业学校晋升交通技师学院，鼓励各县（市）加快发展中等职业教育，培养更多更好的技术技能型人才。完善公共卫生服务体系，做实家庭医生签约服务，完成乡村医生全员培训。全面推进县域综合医改。加快市中心医院改造提升，推进市中心医院新区医院、市中医院东区分院加快建设。发展体育事业，广泛开展全民健身活动。加强食品、疫苗等安全监管，让群众吃得更放心、用药更安全。推进新时代文明实践中心建设，加快媒体融合发展。持续实施文化惠民工程，开展舞台艺术送基层、公益电影放映等活动，繁荣艺术创作，开展“书香周口”读书活动，实现村级综合性文化服务中心全覆盖。创新社会治理。学习推广“枫桥经验”，全面加强基层社会治理。开展阳光信访、责任信访、法治信访，促进人民群众合理合法利益诉求及时、就地解决。加强法治宣传教育和法律服务。深化平安周口创建，加强社会治安防控体系建设，依法打击各类违法犯罪活动，推动“扫黑除恶”专项斗争纵深发展。扎实做好庆祝中华人民共和国成立70周年安保等工作，维护社会大局和谐稳定。

加强国防教育、国防动员和国防后备力量建设，支持国防和军队改革，支持驻周解放军、武警部队、预备役部队、民兵和人民防空建设，推进军民融合发展，深入开展双拥共建，加强退役军人服务保障体系建设，巩固军政军民团结，积极争创全国双拥模范城。全面贯彻党的民族政策和宗教工作基本方针，依法加强宗教事务管理，促进民族团结、宗教和睦。支持工会、共青团、妇联等人民团体更好发挥桥梁纽带作用。加强统计工作，搞好第四次全国经济普查。做好海关、公积金、史志、档案、气象、地震、邮政、外事侨务、社科研究、应急救援、援疆等工作。

三、全面加强政府自身建设

2019年，经济社会发展任务重、挑战多、要求高。要全面完成确定的目标任务，进一步把难得的发展机遇转化为周口大建设、大发展、大变化的生动实践，我们必须注重加强自身建设，全面提高履职能力和服务水平，对人民负责、受人民监督、让人民满意。

强化党的领导。旗帜鲜明把党的政治建设放在首位，认真贯彻落实习近平新时代中国特色社会主义思想，主动对标、自动看齐，增强“四个意识”，坚定“四个自信”，做到“两个维护”，始终在政治立场、政治方向、政治原则、政治道路上同以习近平同志为核心的党中央保持高度一致。切实把党的领导贯彻和体现到政府工作的各方面、全过程，自觉服从市委领导，对市委的决策部署，坚定坚决、不折不扣、落实落细。

强化依法行政。牢固树立法治理念，始终把政府工作全面纳入法治轨道，坚持依宪施政、依法行政、依法办事。健全行政决策机制，落实公众参与、专家论证、风险评估、合法性审查和集体讨论决定等重大决策程序，进一步提高政府决策科学化、民主化、法治化水平。认真执行市人大及其常委会的决议决定，自觉接受人大的法律监督、工作监督和政协的民主监督，认真办理人大代表议案、建议和政协提案。支持民主党派、工商联、无党派人士参政议政。深化政务公开，主动回应社会关切，接受社会和舆论监督。规范行政执法，防止任性用权，纠正粗暴执法，决不允许以权代法、以权压法、逐利违法、徇私枉法。

强化责任担当。始终坚持发展第一要务不动摇，贯彻新发展理念不折腾，推动高质量发展不懈怠，担起该担的责，做好该做的事。锐意进取、高树标尺，推动各项工作创优争先。敢于担当责任，勇于直面困难，不回避矛盾，不惧怕挑战，结合实际创造性开展工作，确保抓一项成一项、干一件成一件。

强化务实重干。面对周口千万人民的期盼，我们将把精力用在大干苦干实干上，真正靠实干让人民过上更加美好的生活。坚持以上率下、上率下行，实实在在当好千万人民的“长工”。对上级的部署、认准的事情、议定的决策，雷厉风行、加快节奏，以钉钉子精神确保工作落到实处。按照干部作风大整顿要求，着力解决懒政怠政、为官不为等问题，为基层减负松绑，坚决防止和克服形式主义、官僚主义，打通服务群众的“最后一公里”。

强化清正廉洁。严格落实全面从严治党主体责任和“一岗双责”，认真贯彻落实中央八项规定及其实施细则精神，推动政府系统党风廉政建设向纵深发展。厉行勤俭节约，反对铺张浪费，压减“三公”经费和一般性支出，用政府的紧日子换来人民群众的好日子。按照“四化三查”要求，规范制约政府行为，做到清白做人、干净做事。严格执行廉洁自律准则和纪律处分条例，筑牢思想防线，保持清醒头脑，始终敬畏人民、敬畏组织、敬畏法纪，努力实现干部清正、政府清廉、政治清明。

各位代表！

幸福是奋斗出来的！周口的路子，是闯出来的；周口的建设，是干出来的；周口的事业，是拼出来的。周口的发展，需要我们上下同心、携手奋进，抓紧每一天、干好每件事。让我们更加紧密地团结在以习近平同志为核心的党中央周围，在省委、省政府和市委的坚强领导下，拿出众人拾柴的心劲，逢山开路的闯劲，甩开膀子的干劲，创优争先的拼劲，跑好“接力赛”，走好“长征路”，为打造“满城文化半城水，内联外通达江海”的中原港城作出更大贡献，以优异成绩庆祝中华人民共和国成立70周年！
